# Improvements in Diabetic Neuropathy and Nephropathy After Bariatric Surgery: a Prospective Cohort Study

**DOI:** 10.1007/s11695-020-05052-8

**Published:** 2020-10-26

**Authors:** Safwaan Adam, Shazli Azmi, Jan H. Ho, Yifen Liu, Maryam Ferdousi, Tarza Siahmansur, Alise Kalteniece, Andrew Marshall, Shaishav S. Dhage, Zohaib Iqbal, Yvonne D’Souza, Salim Natha, Philip A. Kalra, Rachelle Donn, Basil J. Ammori, Akheel A. Syed, Paul N. Durrington, Rayaz A. Malik, Handrean Soran

**Affiliations:** 1 Faculty of Biology, Medicine and Health, University of Manchester, Manchester, UK; 2 The Christie NHS Foundation Trust, Manchester, UK; 3 Manchester University NHS Foundation Trust, Manchester, UK; 4 Wrightington, Wigan and Leigh NHS Foundation Trust, Wigan, UK; 5 Salford Royal NHS Foundation Trust, Salford, UK; 6 Weill-Cornell Medicine-Qatar, Doha, Qatar

**Keywords:** Small nerve fibre, Neuropathy, Obesity, Type 2 diabetes, Bariatric surgery, Microvascular, Retinopathy, Nephropathy

## Abstract

**Purpose:**

There are limited data on the impact of bariatric surgery on microvascular complications of type 2 diabetes (T2D), particularly diabetic neuropathy. We assessed microvascular complications (especially neuropathy) in obese patients with T2D before and 12 months after bariatric surgery.

**Materials and Methods:**

This was a prospective observational cohort study. Measurements of neuropathy symptom profile (NSP), neuropathy disability score (NDS), vibration (VPT), cold (CPT) and warm (WPT) perception thresholds, nerve conduction studies (NCS) and corneal confocal microscopy (CCM) to quantify corneal nerve fibre density (CNFD), branch density (CNBD) and fibre length (CNFL); urinary albumin/creatinine ratio (uACR), estimated glomerular filtration rate (eGFRcyst-creat) and retinal grading were taken.

**Results:**

Twenty-six (62% female; median age 52 years) obese patients with T2D were recruited. Body mass index (BMI) (47.2 to 34.5 kg/m^2^; *p* < 0.001) decreased post-operatively. There were improvements in CNFD (27.1 to 29.2/mm^2^; *p* = 0.005), CNBD (63.4 to 77.8/mm^2^; *p* = 0.008), CNFL (20.0 to 20.2/mm^2^; *p* = 0.001), NSP (3 to 0/38; *p* < 0.001) and eGFRcyst-creat (128 to 120 ml/min; *p* = 0.015) post-bariatric surgery. Changes in (Δ) triglycerides were independently associated with ΔCNFL (*β* = − 0.53; *p* = 0.024) and Δsystolic blood pressure (β = 0.62;*p* = 0.017), and %excess BMI loss (*β* = − 0.004; *p* = 0.018) were associated with ΔeGFRcyst-creat. There was no significant change in NDS, VPT, CPT, WPT, NCS, uACR or retinopathy status. Glomerular hyperfiltration resolved in 42% of the 12 patients with this condition pre-operatively.

**Conclusion:**

Bariatric surgery results in improvements in small nerve fibres and glomerular hyperfiltration in obese people with T2D, which were associated with weight loss, triglycerides and systolic blood pressure, but with no change in retinopathy or uACR at 12 months.

**Electronic supplementary material:**

The online version of this article (10.1007/s11695-020-05052-8) contains supplementary material, which is available to authorized users.

## Introduction

Obesity is a major contributor to the epidemic of type 2 diabetes (T2D) and much of the health and economic burden of T2D relates to its microvascular and macrovascular complications. Bariatric surgery is an effective and durable treatment for the remission of T2D [1] and has long-term benefits for incident major macrovascular and microvascular events. However, there are few detailed studies assessing early outcomes in relation to microvascular complications, particularly neuropathy.

A previous health record-based retrospective cohort study showed that remission of T2D after bariatric surgery conferred protection against the development of microvascular complications even after relapse of T2D [2]. Similarly, in a large retrospective cohort study, 5-year incident microvascular complications were reduced by 78% after bariatric surgery. Because microvascular complications were defined by the end-stage outcomes of amputation, laser eye surgery or blindness and dialysis [3], the benefits of bariatric surgery have not been reported until at least 5 years of follow-up [4].

More detailed short-term studies of microvascular outcomes have however reported conflicting results. A recent meta-analysis of seven controlled studies found that bariatric surgery prevented the development of diabetic retinopathy but did not impact on progression or regression of retinopathy [5]. With regard to nephropathy, urinary albumin/creatinine ratio (uACR) was reduced, and both glomerular hypo- and hyperfiltration improved after bariatric surgery [6, 7]. Data relating to diabetic neuropathy are contradictory, as there are reports of worsening symptomatic neuropathy, attributed to nutritional deficiencies [8] and a 33% incidence of neuropathic pain following bariatric surgery [9]. In a prospective cohort study of 20 participants undergoing bariatric surgery, there were significant improvements in the neuropathy symptom and disability scores after 6 months [10]. However, Miras et al. found no significant improvement in radial, sural and peroneal nerve conduction velocities or amplitudes 12 months after bariatric surgery [11].

Our aim was to assess the effect of bariatric surgery over 12 months on microvascular complications in a cohort of obese patients with T2D. We performed detailed neuropathy phenotyping, especially, using corneal confocal microscopy (CCM) as it has been shown to detect early small fibre repair following simultaneous pancreas and kidney transplantation in type 1 diabetes [12].

## Methods

### Study Design and Patient Recruitment

We prospectively studied 26 obese patients with T2D undergoing bariatric surgery at a Tier 4 specialist weight management service in the North West of England. Assessments were undertaken before and 12 months after bariatric surgery. None of the patients that enrolled were lost to follow-up. Participants with a history of corneal trauma, surgery or disease were excluded from the study. Patients with a history of retinal, renal or neuropathic disease not due to T2D were also excluded. Ethical approval was sought and granted by the Central Manchester Research and Ethics Committee with all patients providing informed consent before study participation. Patients were recruited from the pre-operative clinic between October 2014 and January 2016, and all study assessments were completed by April 2017*.*

### Surgery

Laparoscopic Roux-en-Y gastric bypass (RYGB) involved the fashioning of a short 5-cm vertical gastric pouch based on the lesser curvature of the stomach and constructed over a 40 French orogastric tube using staplers. An ante-colic ante-gastric Roux-en-Y gastrojejunostomy was fashioned with the bilio-enteric limb measuring 50–100 cm and the alimentary limb measuring 75–150 cm depending on the patient’s BMI. The jejunojejunostomy was fashioned using either linear staplers or intracorporeal suturing technique, and the gastrojejunostomy was constructed either using a side-to-side linear stapler (45 mm) or intracorporeally sutured in an end-to-side manner over 40 French orogastric tube depending on each surgeon’s preference.

Laparoscopic sleeve gastrectomy (LSG) involved the construction of a vertical gastric sleeve over a 40 French orogastric tube starting 4–6 cm from the pylorus and ending approximately 1 cm lateral to the angle of His using staplers.

#### Clinical and Laboratory Assessment

Body mass index (BMI) was assessed at each visit, and the percent excess BMI loss (%EBMIL) was calculated using the difference in proportionate change in BMI in excess of the upper limit of normal BMI of 24.9 kg/m^2^ before and after bariatric surgery. Blood pressure was measured after resting in a seated position for 5 min, using an Omron HEM 705-CP semiautomatic oscillometric recorder.

Fasting venous blood samples and early morning urine samples were collected at each visit. Glycated haemoglobin (HbA1c), serum creatinine, uACR, total cholesterol, triglycerides and high-density lipoprotein cholesterol were measured in the biochemistry laboratory at Manchester University Hospitals NHS Foundation Trust using routine methods. Serum cystatin C was assayed in the Cardiovascular Research Group Lab at the University of Manchester using immunoturbidimetric assays with a Cobas Mira analyser (Horiba ABX Diagnostics, Nottingham, UK). The laboratories participated in the UK National External Quality Assessment Service (UKNEQAS, Birmingham, UK) for quality control of general blood chemistry and urinary chemistry. Low-density lipoprotein cholesterol was calculated using the Friedewald formula.

Complete remission from T2D was classified with an HbA1c below 6.0% (42 mmol/mmol) and no active pharmacological therapy, as per the American Diabetes Association consensus statement [13].

### Diabetic Neuropathy

The neuropathy symptom profile questionnaire consists of 38 questions which assess symptoms of sensory, motor and autonomic neuropathy. The neuropathy disability score includes an assessment of pinprick, temperature sensation (using hot and cold rods), vibration sensation (tuning fork) and ankle reflexes. A score between 0 and 2 is considered normal, 3–5 is mild neuropathy, 6–8 is moderate neuropathy and 9–10 is severe neuropathy. A Neurothesiometer (Horwell, Scientific Laboratory Supplies, Wilford, Nottingham, UK) was used to determine the vibration perception threshold. Cold and warm thermal thresholds were determined using the TSA-II NeuroSensory Analyser (Medoc Ltd., Ramat Yishai, Israel) on the S1 dermatome of the left foot. Nerve conduction studies were performed by a consultant neurophysiologist using a “Keypoint” system (Dantec Dynamics Ltd., Bristol, UK) equipped with a DISA temperature regulator to maintain limb temperature between 32 and 35 °C. Deep breathing heart rate variability was measured using an ANX 3.0 autonomic nervous system monitoring device (ANSAR Medical Technologies, Philadelphia, PA, USA).

### Corneal Confocal Microscopy

Corneal confocal microscopy (Heidelberg Retinal Tomograph III Rostock Cornea Module, Heidelberg Engineering GmbH, Heidelberg, Germany) comprising six non-overlapping corneal images per patient (three per eye) from the centre of the cornea was performed using our established protocol [14]. Three corneal nerve parameters were manually quantified using CCMetrics (The University of Manchester, Manchester, UK): corneal nerve fibre density (CNFD), the total number of major nerves/mm^2^ of corneal tissue; corneal nerve branch density (CNBD), the number of branches emanating from the major nerve trunks/mm^2^ of corneal tissue and corneal nerve fibre length (CNFL), the total length of all nerve fibres and branches (mm/mm^2^) within the area of corneal tissue.

### Diabetic Kidney Disease

The CKD-EPI (2012) equation (combining cystatin C and creatinine, unadjusted for body surface area; CKD-EPIcyst-creat) was used to determine estimated glomerular filtration rate (eGFR). Glomerular hyperfiltration was defined as an eGFR > 125 ml/min in keeping with a recent meta-analysis by Li et al. which analysed changes in eGFR post-bariatric surgery [15]. Albuminuria was defined as uACR > 3.5 mg/mmol in women and > 2.5 mg/mmol in men.

### Diabetic Retinopathy

Two (optic disc and macula centred) 45-degree digital retinal images were used to grade retinopathy from the NHS Diabetic Eye Screening Programme (NHS DESP), as part of the patient’s routine diabetes clinical care before and after surgery. Nationally accredited screeners classified diabetic retinopathy status according to the NHS DESP feature-based grading classification [16]. For quality assurance, an independent ophthalmologist also graded images without prior knowledge of the pre-existing grading.

### Statistical Analysis

SPSS for Mac (Version 23.0, IBM SPSS Statistics, Armonk, NY: IBM Corp.) and GraphPad Prism (Version 7.00, GraphPad Software, La Jolla, CA, USA) were used for analysis of data. Tests for normality were done using the Shapiro-Wilk test, visualisation of histograms and Q-Q plots. To compare means pre- and post-bariatric surgery, paired *t* tests were used for normally distributed variables; Wilcoxon matched pairs test was used for non-parametric variables and McNemar’s test for categorical variables. Tests for relationships between percentage changes (from baseline to 12 months) in variables utilised Pearson’s coefficient for parametric data and Spearman’s coefficient for non-parametric data. Multifactorial linear regression was used to assess for associations between percentage changes in variables. Variables chosen for regression models were based on predicted influential factors. A *p* value of < 0.05 was considered as statistically significant. The a priori estimated sample size required to assess for changes in CCM parameters (the main outcome of interest) was 23 patients to achieve an alpha of 0.05 and statistical power of 80%. These calculations were based on pilot data obtained for a different project in a similar cohort (unpublished data on file).

## Results

### Participant Characteristics

We assessed 26 participants at baseline and 12 months after bariatric surgery (RYGB (*n* = 21), LSG (*n* = 5) (Table 1).

**Table 1:** Clinical and metabolic variables pre and post-bariatric surgery

Variable	Baseline (*n* = 26)	12 months (*n* = 26)	*p* value
Age	52 (10)		
Female (%)	16 (62%)		
Diabetes duration (years)	6 (3–12)		
Insulin treatment	8 (31%)	2 (8%)	**0.041**
ACE-I or ARB treatment	18 (69%)	11 (42%)	**0.023**
Statin treatment	19 (73%)	13 (50%)	**0.041**
Weight (kg)	137 (120–152)	93 (85–117)	**< 0.001**
BMI (kg/m^2^)	47.2 (43.0–57.0)	34.5 (30.0–38.4)	**< 0.001**
Systolic BP (mmHg)	134 (15)	119 (15)	**< 0.001**
Diastolic BP (mmHg)	75 (73)	70 (11)	**0.016**
HbA1c (%) (mmol/mol)	6.9 (6.4–8.6) 52 (46–71)	5.5 (5.3–6.0) 37 (34–42)	**< 0.001**
Total cholesterol (mg/dL) (mmol/l)	144 (28.6) 3.72 (0.74)	162 (36.7) 4.20 (0.95)	**0.035**
Triglycerides (mg/dl) (mmol/l)	134 (81.4–165) 1.51 (0.92–1.86)	100 (77.0–132) 1.13 (0.87–1.49)	0.071
HDL-C (mg/dl) (mmol/l)	33.2 (29.7–39.0) 0.86 (0.77–1.01)	44.0 (38.6–50.6) 1.14 (1.00–1.31)	**< 0.001**
LDL-C (mg/dl) (mmol/l)	81.9 (23.9) 2.12 (0.62)	93.8 (35.1) 2.43 (0.91)	0.198

Data presented as mean (SD) or median (interquartile range). Statistically significant variables are denoted in bold *(p < 0.05*). There were significant (*p* < 0.05) reductions in the use of medication, weight, BMI, blood pressure, HbA1c, total cholesterol and increase in HDL cholesterol post-operatively

*ACE-I* angiotensin-converting enzyme inhibitors, *ARB* angiotensin II receptor blockers, *BMI* body mass index, *BP* blood pressure, *HbA1c* glycated haemoglobin, *HDL-C* high-density lipoprotein cholesterol, *LDL-C* low-density lipoprotein cholesterol

There was a significant reduction in BMI (*p* < 0.001), with a mean %EBMIL of 61 ± 16%. Complete remission of type 2 diabetes occurred in 21 out of 26 (81%) participants (*p* = 0.0001). There were significant reductions in patients using insulin (*p* = 0.04), angiotensin-converting enzyme inhibitors or angiotensin II receptor blockers (ACEi/ARB) (*p* = 0.02) and statins (*p* = 0.04) (Table 1). There were significant reductions in systolic (*p* < 0.001) and diastolic (*p* < 0.02) blood pressures and HbA1c (*p* < 0.001) and an increase in total cholesterol (p = 0.04) and high-density lipoprotein cholesterol (*p* < 0.001) (Table 1).

### Diabetic Neuropathy

Neuropathy symptom profile showed a significant improvement (*p* < 0.001). Based on the neuropathy disability score, 6/26 participants had diabetic neuropathy at baseline (4 mild, 1 moderate, 1 severe) and showed a non-significant (*p* = 0.07) trend for improvement after bariatric surgery (Table 2). Quantitative sensory testing showed no significant improvements in vibration, cold or warm perception thresholds 12 months after bariatric surgery (Table 2).

**Table 2:** Microvascular assessments pre- and post-bariatric surgery

Parameter	Baseline (*n* = 26)	12 months (*n* = 26)	*p*
*Neuropathy assessment*			
NSP (x/38)	3 (0–5)	0 (0–1)	**< 0.001**
NDS (x/10)	1 (0–3)	0 (0–2)	0.068
VPT (volts)	14.2 (7.06)	13.6 (7.11)	0.969
CPT (°C)	25.7 (20.0–28.1)	26.3 (22.0–28.3)	0.702
WPT (°C)	40.0 (3.98)	41.3 (4.76)	0.093
DB-HRV (beats/min)	15 (12–22)	14 (11–20)	0.670
CNFD (no./mm^2^)	27.1 (20.8–30.2)	29.2 (25–34.9)	**0.005**
CNBD (no./mm^2^)	63.4 (35.1)	77.8 (35.5)	**0.008**
CNFL (mm/mm^2^)	20.0 (15.8–22.7)	20.2 (18.3–23.8)	**0.001**
*Renal assessment*			
uACR (mg/mmol)	1.00 (0.57–1.71)	0.50 (0.34–1.00)	0.103
sCreat (μmol/l) (mg/dl)	77 (27) 0.87 (0.31)	66 (17) 0.75 (0.19)	**< 0.001**
sCysC (mg/l)	0.9 (0.72–1.03)	0.87 (0.79–1.11)	0.348
eGFR (ml/min)	128 (26)	120 (23)	**0.015**

Data are presented as mean (SD) or median (interquartile range). Variables in bold are statistically significant (*p < 0.05*)

There was a significant improvement in the NSP, CNFD, CNBD and CNFL (*p* < 0.01). Other variables showed a non-significant trend towards improvement

*NSP* neuropathy symptom profile, *NDS*, neuropathy disability score, *VPT* vibration perception threshold, *CPT* cold perception threshold, *WT* warm perception threshold, *DB-HRV* deep breathing heart rate variability, *CNFD* corneal nerve fibre density, *CNBD* corneal nerve branch density, *CNFL* corneal nerve fibre length, *ACR* albumin/creatinine ratio, *sCreat* serum creatinine, *sCysC* serum cystatin C, *eGFR* estimated glomerular filtration rate

#### Corneal Confocal Microscopy

**Fig. 1:**
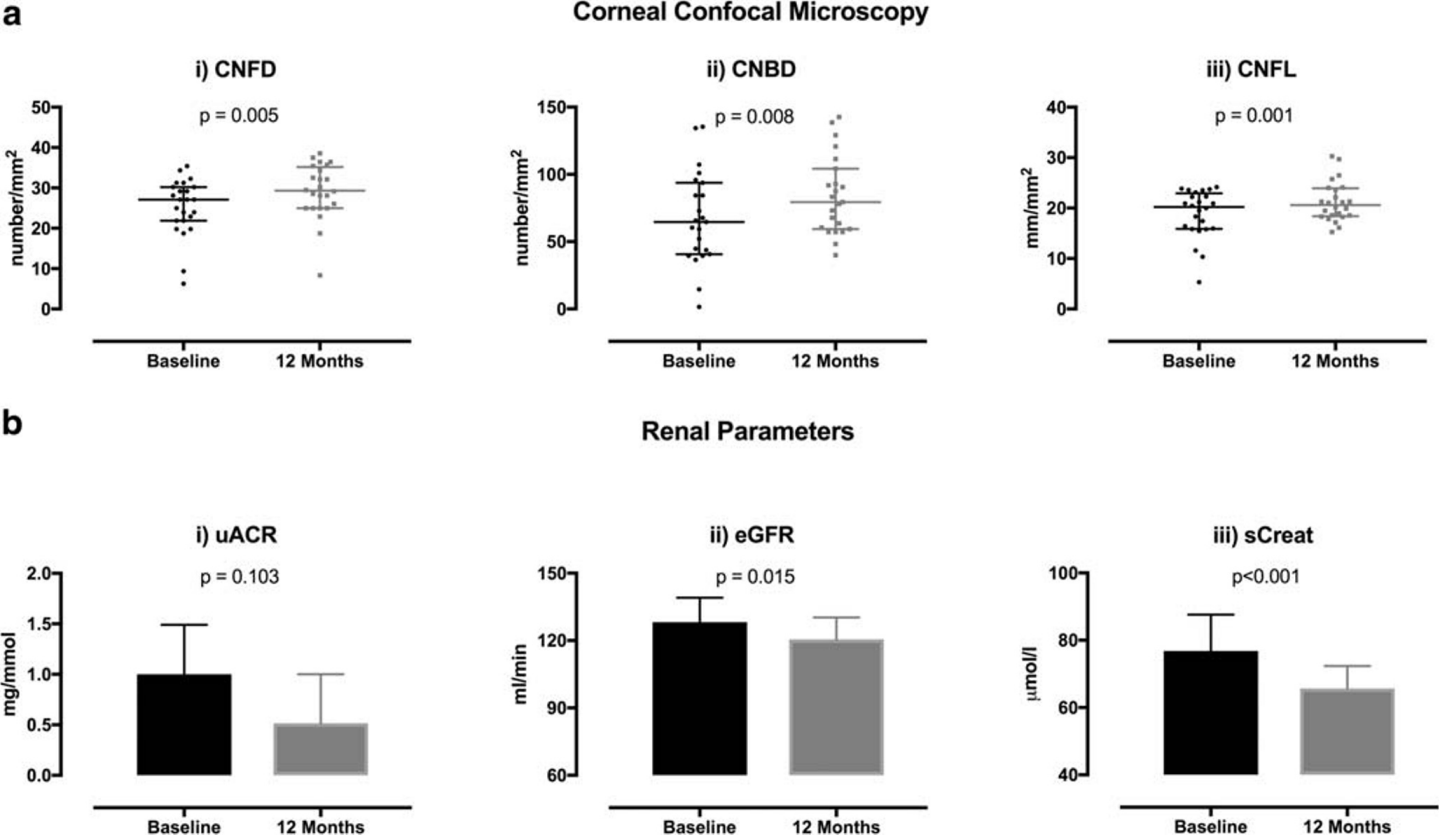
Microvascular outcome parameters before and after bariatric surgery. Figure 1 depicts significant improvements in the CNFD, CNBD and CNFL from baseline to 12 months after bariatric surgery. There were also significant reductions in the eGFR, serum creatinine and a change in uACR (non-significant). **a** Corneal confocal microscopy. **a** (i) Corneal nerve fibre density before and after surgery, (ii) corneal nerve branch density before and after surgery, (iii) corneal nerve fibre length before and after surgery. **b** Renal parameters. **b** (i) urinary albumin/creatinine ratio before and after surgery, (ii) estimated glomerular filtration rate before and after surgery, (iii) serum creatinine before and after surgery. CNFD, corneal nerve fibre density; CNBD, corneal nerve branch density; CNFL, corneal nerve fibre length; uACR, urinary albumin/creatinine ration; eGFR, estimated glomerular filtration rate; sCreat, serum creatinine

**Fig. 2:**
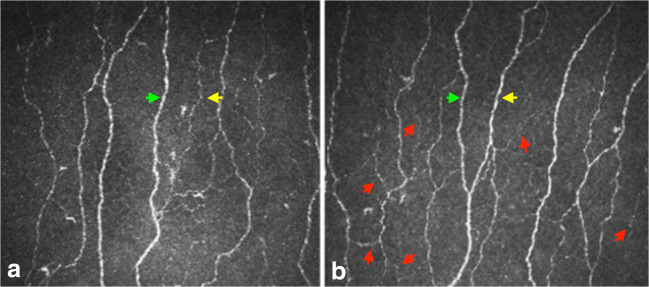
Example corneal confocal microscopy image in a participant. This image obtained using corneal confocal microscopy shows an improvement in the corneal nerve morphology from pre- (**a**) to post- (**b**) bariatric surgery. In the post-operative image (**b**), there are more nerves seen, and the red arrows depict small nerve fibre branches indicative of regeneration. The green arrows illustrate a main nerve fibre and the yellow arrows depict nerve fibre branches

Corneal nerve fibre density (*p* < 0.005), nerve branch density (*p* = 0.008) and nerve fibre length (*p* = 0.001) improved significantly after bariatric surgery (Table 2, Figs. 1 and 2).

#### Cardiac Autonomic Function

There was no change in deep breathing heart rate variability after bariatric surgery (Table 2).

#### Nerve Conduction Studies

Nerve conduction studies was performed in 9/26 patients before and after surgery with no significant difference between participants who did and did not undergo nerve conduction studies in relation to baseline BMI, blood pressure, diabetes duration and measures of neuropathy. There was no significant change in sural nerve latency, amplitude, conduction velocity, peroneal nerve latency, amplitude and velocity and radial nerve amplitude and velocity (Supplementary Table 1).

### Diabetic Retinopathy

At baseline 5 (19%) participants had background retinopathy (R1); it improved in one patient with no deterioration in any of the participants. One patient had maculopathy at baseline, which had resolved at follow up without any specific treatment for the maculopathy.

### Diabetic Kidney Disease

There was a significant decrease in serum creatinine (*p* < 0.001), no change in serum cystatin C (Table 2) and a significant decrease in eGFR (*p* < 0.02) after bariatric surgery (Table 2; Fig. 1). Pre-operatively 12 (46%) patients fulfilled the criteria for glomerular hyperfiltration, and the eGFR fell below 125 ml/min in 5 participants after surgery (*p* = 0.07). There was no significant decrease in uACR (*p* = 0.10) (Table 2; Fig. 1). Seven patients stopped ACEi therapy due to normalisation of blood pressure. There were no significant differences in baseline or post-operative results between patients who were or were not being treated with ACEi.

### Relationships Between Changes in Metabolic Parameters and Microvascular Outcomes

There was no correlation between the change in BMI, HbA1c and lipids and change in microvascular complications. Changes in systolic blood pressure correlated with changes in eGFR (*r* = 0.43; *p* = 0.027).

Multifactorial regression was used to assess for the potential influence of changes in metabolic measures on microvascular disease outcomes (Table 3). There was a significant association between change (Δ) in triglycerides (*β* = − 0.53; *p* = 0.024) and change in CNFL and %EBMIL (*β* = − 0.004; *p* = 0.018) and Δsystolic blood pressure (*β* = 0.62; *p* = 0.017) with ΔGFR.

**Table 3:** Association between changes in metabolic variables and changes in microvascular outcome measures after bariatric surgery

Variable	Coefficient	95% confidence interval	*p*
Percentage change in CNFL			
Pre-operative diabetes duration	0.002	− 0.009 to 0.013	0.682
ΔHbA1c	0.158	− 0.177 to 0.492	0.331
ΔSBP	0.497	− 0.071 to 1.065	0.082
ΔTriglycerides	**− 0.135**	**− 0.250 to − 0.020**	**0.024**
%EBMIL	0.004	0.000 to 0.008	0.05
Percentage Change in eGFR			
Pre-operative diabetes duration	0.002	− 0.007 to 0.011	0.637
ΔHbA1c	− 0.093	− 0.383 to 0.196	0.502
ΔSBP	**0.619**	**0.127 to 1.111**	**0.017**
ΔTriglycerides	− 0.066	− 0.165 to 0.034	0.180
%EBMIL	**− 0.004**	**0.001 to 0.007**	**0.018**
Percentage change in urinary ACR			
Pre-operative diabetes duration	**0.248**	**0.042 to 0.453**	**0.021**
ΔHbA1c	− 4.108	− 10.456 to 2.241	0.188
ΔSBP	− 3.282	− 14.069 to 7.505	0.526
ΔTriglycerides	− 0.791	− 2.971 to 1.390	0.452
%EBMIL	0.037	− 0.034 to 0.108	0.286
Percentage change in CNFD			
Pre-operative diabetes duration	0.006	− 0.082 to 0.094	0.884
ΔHbA1c	0.111	− 2.643 to 2.864	0.932
ΔSBP	− 1.047	− 5.709 to 3.615	0.638
ΔTriglycerides	− 0.204	− 1.145 to 0.738	0.650
%EBMIL	0.020	− 0.013 to 0.053	0.223
Percentage change in CNBD			
Pre-operative diabetes duration	− 0.040	− 0.093 to 0.012	0.121
ΔHbA1c	0.480	− 0.955 to 1.914	0.485
ΔSBP	0.082	− 2.375 to 2.539	0.944
ΔTriglycerides	− 0.070	− 0.599 to 0.459	0.782
%EBMIL	− 0.001	− 0.02 to 0.017	0.879

Multifactorial linear regression assessing relationships between percentage changes in microvascular outcomes and percentage change in metabolic variables. Diabetes duration relates to pre-operative duration of diabetes. Variables in bold text are statistically significant (*p < 0.05*)

*CNFL* corneal nerve fibre length, *eGFR* estimated glomerular filtration rate, *ACR* albumin/creatinine ratio, *CNFD* corneal nerve fibre density, *CNBD* corneal nerve branch density, *%EBMIL* percentage excess body mass index loss (proportionate change in excess of BMI of > 25 kg/m^2^)

Δ = percentage change from baseline

### Comparison Between Surgical Procedures

There were no significant differences in the baseline clinical characteristics between the 21 patients who underwent RYGB and the 5 patients who underwent LSG (Supplementary Tables 2 and 3). Post-operatively, patients who underwent RYGB had significantly lower triglyceride levels. Although post-operatively, BMI and systolic blood pressure reduced significantly in both groups (RYGB and LSG), significant improvements in ACE-i use, HbA1c, serum triglycerides and high-density lipoprotein cholesterol were only seen in RYGB patients (Supplementary Table 2). Complete diabetes remission occurred in 17 RYGB patients (81%) and 4 (80%) LSG patients (*p* = 0.97).

There were no significant differences in baseline or post-operative microvascular disease parameters in both groups of patients. Although the neuropathy symptom profile improved significantly in both groups, significant improvements in the corneal nerve fibre density, corneal nerve branch density, corneal nerve fibre length and eGFR occurred only in patients who underwent RYGB (Supplementary Table 3). There were no significant procedure-related differences in the pre- to post-operative percentage changes in CCM parameters (Supplementary Fig. 1).

## Discussion

This is the first study to show an improvement in diabetic neuropathy and diabetic kidney disease but not diabetic retinopathy, 12 months after bariatric surgery in obese patients with type 2 diabetes. There was a marked improvement in BMI, blood pressure, HbA1c and lipid profile, in keeping with previous studies [1] and a 77% remission rate of type 2 diabetes in our cohort.

Previous reports have shown a small fibre neuropathy in obese patients without diabetes [17] and in participants with impaired glucose tolerance [18], particularly those who develop T2D [19]. Furthermore, a reduction in corneal nerve fibre length has been associated with age, HbA1c and high-density lipoprotein cholesterol [20]. The evaluation of small fibre damage was a major outcome in our study as CCM and skin biopsy have shown that small nerve fibre pathology precedes abnormality in quantitative sensory testing and nerve conduction studies in patients with sub-clinical diabetic neuropathy [21]. We have also shown that corneal nerve fibre length is reduced in diabetic patients without microalbuminuria [22] or retinopathy [23] and predicts the development of diabetic neuropathy [24] and retinopathy [25]. This indicates that CCM can detect early small fibre damage [26].

This study shows an improvement in the neuropathy symptom profile, which takes into account sensory, motor and autonomic symptoms, in keeping with the results of the DiaSurg1 study [10]. The DiaSurg1 study also found an improvement in the neuropathy disability score, driven by changes in vibration perception and Achilles reflexes [10], implying large fibre benefits. However, in the present study, we found no improvement in the neuropathy disability score, vibration perception threshold or nerve conduction studies. This confirms the study by Miras et al., which also found no significant improvement in radial, sural and peroneal nerve conduction velocities or amplitudes after bariatric surgery [11].

In the DiaSurg1 study, there was no change in temperature perception or pinprick thresholds [10], suggesting no impact on small fibres. Whilst we also found no improvement in cold and warm temperature thresholds or deep breathing heart rate variability, there was a significant improvement in corneal nerve morphology. CCM has previously been used to show corneal nerve fibre repair, despite no change in neuropathy symptoms and deficits, neurophysiology, quantitative sensory testing and skin biopsy in patients with type 1 diabetes following simultaneous pancreas and kidney transplantation [12, 27]. A novel first-in-class peptide ARA290 (Cibinetide) which blocks inflammation has been shown to improve corneal nerve fibre density and length in patients with sarcoidosis-related neuropathy [28, 29] and T2DM [30], which was paralleled by an improvement in pain scores and functional outcomes. In a 12-month trial of seal oil omega-3 polyunsaturated fatty acid in patients with T1DM, there was a 29% increase in CNFL, with no change in nerve conduction velocity and sensory function [31].

Glomerular hyperfiltration may occur in the earliest phase of nephropathy in patients with type 2 diabetes. However, obesity per se is also associated with increased glomerular filtration. In our cohort, the mean pre-operative eGFR was in keeping with glomerular hyperfiltration [15]. Post-bariatric surgery, there is loss of adipose tissue, muscle mass and body surface area, which can impact upon the glomerular filtration rate. To account for this, our eGFR calculation utilised both changes in creatinine and cystatin C, and the eGFR measurement without indexing to body surface area [32]. Our results show an apparent discordant relationship between serum creatinine and eGFR, as usually a fall in creatinine (as was seen post-operatively) should lead to a rise in eGFR. However, in our study eGFR was reduced after surgery as the calculation method we used accounted for changes in cystatin C and body surface area as opposed to creatinine alone. Indeed, Friedman et al. have shown that CKD-EPIcreat-cyst is the most accurate means of calculating eGFR against measured GFR in a bariatric cohort [32]. The participants in this study on the whole had normal uACR readings, and whilst there was a tendency towards a reduction in this parameter, this did not reach statistical significance. There was no change in retinopathy status in our cohort, which is in keeping with a recent meta-analysis [5].

In the current study, the reduction in triglycerides was associated with an increase in corneal nerve fibre length. Previous reports have shown that hypertriglyceridemia is a risk factor for the development of small fibre neuropathy in diabetic patients [33]. Additionally, there was a direct relationship between the reduction in systolic blood pressure and eGFR, and the inverse relationship between excess BMI loss and ΔeGFR may indicate that weight loss reduces glomerular hyperfiltration, as both obesity and hypertension are risk factors for glomerular hyperfiltration. The change in HbA1c did not significantly influence change in any of the microvascular outcome measures; however, the baseline HbA1c was excellent, and the change in HbA1c was small.

The main strength of the study is the state-of-the-art methods used concurrently to assess retinopathy, nephropathy and particularly neuropathy. We confirm CCM identifies small nerve fibre regeneration following bariatric surgery, demonstrating the utility of CCM as a surrogate end point for the assessment of nerve fibre repair.

The limitations of this study include the small sample size and lack of a matched control group of patients with type 2 diabetes who have not undergone bariatric surgery. Also, due to the small number of patients who underwent sleeve gastrectomy as opposed to gastric bypass, we could not comprehensively test for procedure-specific effects. However, our limited data suggested that in this cohort of patients, there was no significant procedural influence. Nerve conduction studies were only available for 9 participants, primarily due to excess subcutaneous adipose tissue in these patients. Furthermore, the follow-up period in our study was relatively short and prevented us from assessing the impact of weight gain which typically occurs 24 months after bariatric surgery [34]. Therefore, the longer term implications of our findings will need further evaluation.

In conclusion, we show for the first time that bariatric surgery can potentially lead to an early reversal of diabetic neuropathy, particularly small fibre pathology. We also report a beneficial effect on glomerular hyperfiltration, but no impact on albuminuria or retinopathy. These improvements may be driven by an improvement in weight, systolic blood pressure and triglycerides, which warrant further study.

## Electronic Supplementary Materials

Supplementary Figure 1 There were no significant differences between patients who underwent RYGB and LSG in the pre-operative to post-operative percentage changes in corneal nerve parameters. RYGB: Laparoscopic Roux-en-Y Gastric Bypass; LSG: Laparoscopic Sleeve Gastrectomy. (PNG 156 kb)

High Resolution Image (TIFF 2646 kb)

Table S1 (DOCX 14 kb)

Table S2 (DOCX 17 kb)

Table S3 (DOCX 17 kb)
