# Supplementary figures and images for: Improvements in Diabetic Neuropathy and Nephropathy After Bariatric Surgery: a Prospective Cohort Study
Source: Obes Surg. 2020 Oct 26;31(2):554–63. doi: 10.1007/s11695-020-05052-8 (PMC7847862; doi:10.1007/s11695-020-05052-8)

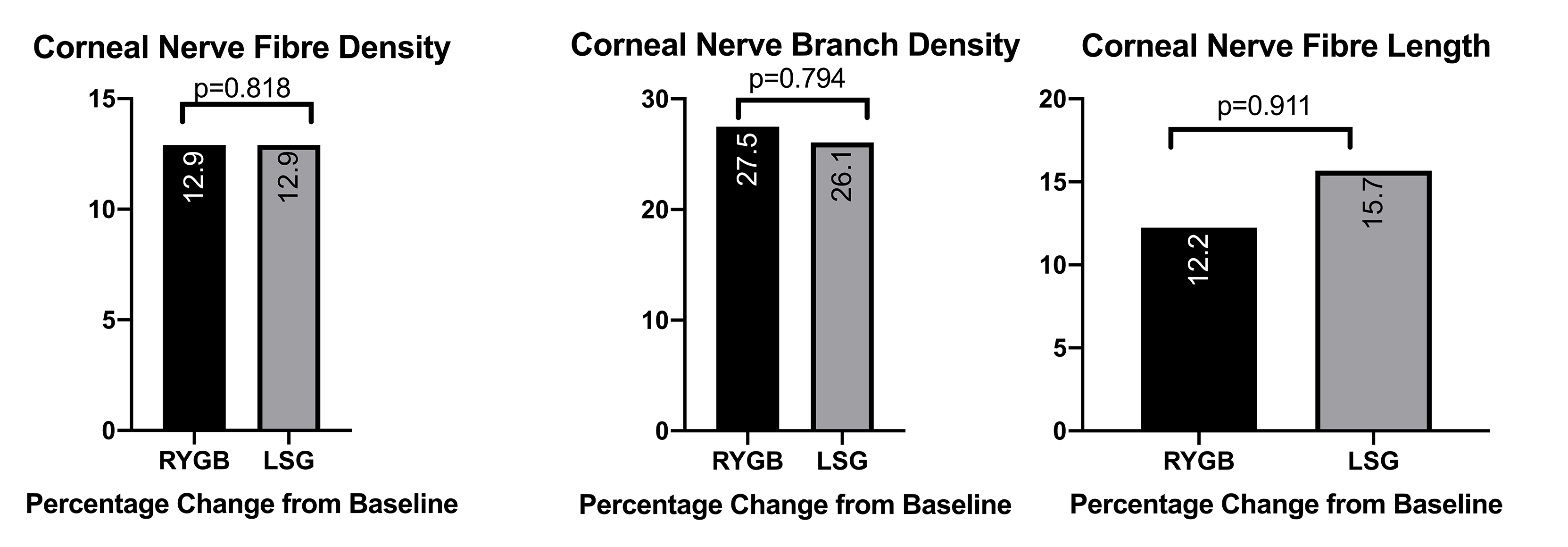

Supplement: Supplementary file 1 — There were no significant differences between patients who underwent RYGB and LSG in the pre-operative to post-operative percentage changes in corneal nerve parameters. RYGB: Laparoscopic Roux-en-Y Gastric Bypass; LSG: Laparoscopic Sleeve Gastrectomy. (PNG 156 kb) [file 11695_2020_5052_Fig3_ESM.png]

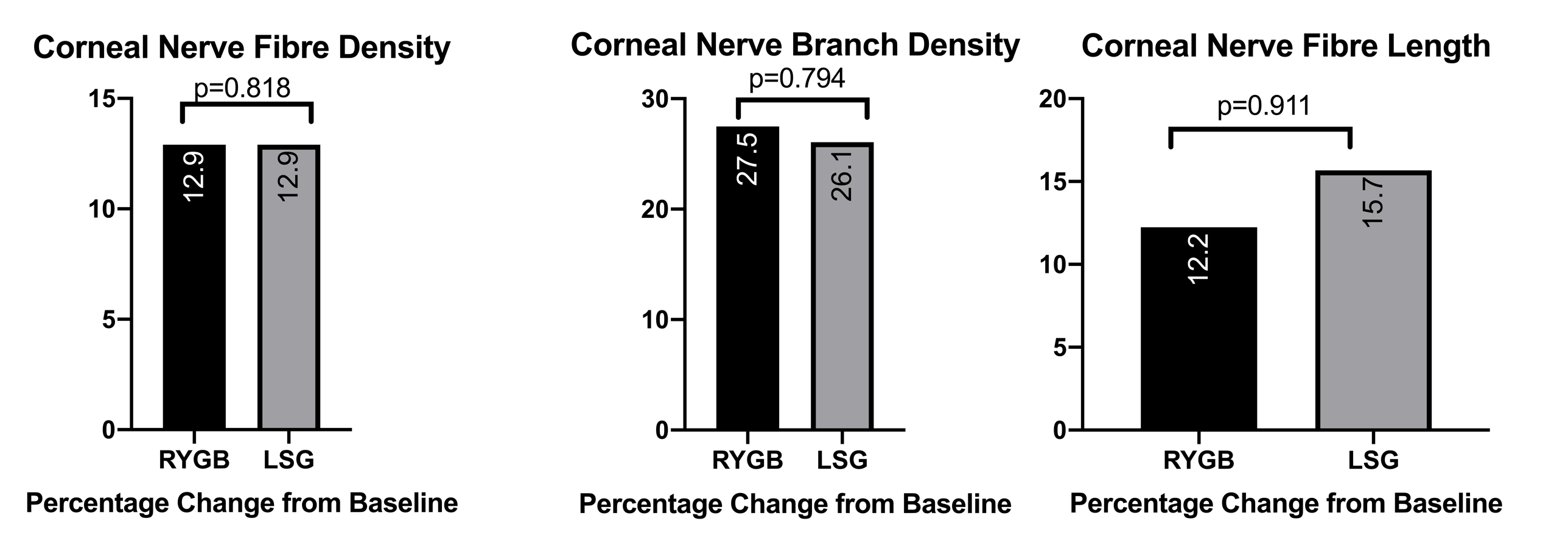

Supplement: Supplementary file 2 — High Resolution Image (TIFF 2646 kb) [file 11695_2020_5052_MOESM1_ESM.tiff]
