# Supplementary material for: Improvements in Diabetic Neuropathy and Nephropathy After Bariatric Surgery: a Prospective Cohort Study
Source: Obes Surg. 2020 Oct 26;31(2):554–63. doi: 10.1007/s11695-020-05052-8 (PMC7847862; doi:10.1007/s11695-020-05052-8)
Supplement: Supplementary file 3 — (DOCX 14 kb) [file 11695_2020_5052_MOESM2_ESM.docx]

Supplementary Table 1. Nerve Conduction Studies pre and post-bariatric surgery (n=9).

| Neurophysiology | Baseline | 12 Months | p-value |
| --- | --- | --- | --- |
| Sural nerve latency (ms) | 2.84 (0.55) | 3.04 (0.32) | 0.384 |
| Sural nerve amplitude (μV) | 8.15 (5.68) | 6.94 (3.84) | 0.820 |
| Sural nerve velocity (m/s) | 49.9 (6.69) | 46.5 (4.04) | 0.380 |
| Peroneal nerve latency (ms) | 4.07 (0.46) | 4.23 (0.42) | 0.221 |
| Peroneal nerve amplitude (mV) | 3.14 (0.94) | 3.08 (1.35) | 0.905 |
| Peroneal nerve velocity (m/s) | 45.6 (5.06) | 45.3 (3.22) | 0.848 |
| Radial nerve amplitude (μV) | 33.2 (11.5) | 34.9 (12.3) | 0.216 |
| Radial nerve velocity (m/s) | 61.4 (3.09) | 59.4 (4.05) | 0.275 |

Data are presented as mean (SD).
