# Supplementary material for: Improvements in Diabetic Neuropathy and Nephropathy After Bariatric Surgery: a Prospective Cohort Study
Source: Obes Surg. 2020 Oct 26;31(2):554–63. doi: 10.1007/s11695-020-05052-8 (PMC7847862; doi:10.1007/s11695-020-05052-8)
Supplement: Supplementary file 4 — (DOCX 17 kb) [file 11695_2020_5052_MOESM3_ESM.docx]

| Variable (n=26) | Baseline |  |  | 12 Months |  |  |
| --- | --- | --- | --- | --- | --- | --- |
|  | RYGB | LSG | p | RYGB | LSG | p |
| Age | 50 (9) | 58 (12) | 0.100 |  |  |  |
| Female (%) | 57% | 80% | 0.617 |  |  |  |
| Diabetes Duration (years) | 6 (4-12.5) | 2 (1.5-10.5) | 0.09 |  |  |  |
| Insulin Treatment | 8/21 (38%) | 0/5 | 0.281 | 2/21 (9.5%)* | 0 | 1.00 |
| ACE-I or ARB | 15/21 (71%) | 3/5 (60%) | 0.628 | 8/21 (38%)* | 3/5 (60%) | 0.620 |
| Statin Treatment | 16/21 (76%) | 3/5 (60%) | 0.588 | 12/21 (57%) | 1/5 (20%) | 0.322 |
| BMI (kg/m^2^) | 50.9 (9.0) | 43.2 (4.2) | 0.08 | 35.1 (6.0)*** | 33.6 (3.3)** | 0.600 |
| Systolic BP (mmHg) | 133 (16) | 136 (11) | 0.687 | 118 (15)*** | 120 (14)* | 0.852 |
| Diastolic BP (mmHg) | 75 (13) | 74 (12) | 0.880 | 70 (12) | 67 (9) | 0.551 |
| HbA1c (%)  (mmol/mol) | 7.3 (6.4-9.3)  56 (46-78) | 6.7 (6.3-7.4)  50 (45-57) | 0.237 | 5.5*** (5.2-6.1)  37*** (33-43) | 5.5 (5.4-6.5)  37 (35-47) | 0.694 |
| Total Cholesterol (mg/dl)  (mmol/l) | 146 (29)  3.78 (0.75) | 135 (29)  3.50 (0.75) | 0.453 | 157 (34)  4.06 (0.89) | 195 (24)*  5.05 (0.63)* | 0.051 |
| Triglycerides (mg/dl)  (mmol/l) | 132 (93)  1.49 (1.05) | 133 (32)  1.50 (0.36) | 0.996 | 81 (22)*  0.92 (0.24)* | 167 (76)  1.89 (0.86) | <0.001 |
| HDL-C (mg/dl)  (mmol/l) | 36 (9)  0.92 (0.24) | 34 (5)  0.88 (0.13) | 0.766 | 45 (10)***  1.17 (0.27)*** | 43 (8)  1.10 (0.21) | 0.609 |
| LDL-C (mg/dl)  (mmol/l) | 83 (23)  2.15 (0.61) | 75 (25)  1.94 (0.64) | 0.498 | 84 (32)  2.18 (0.83) | 120 (26)  3.09 (0.68) | 0.059 |

Supplementary Table 2. Clinical and metabolic variables pre and post-bariatric surgery in patients who underwent Roux-en-Y Gastric Bypass and Laparoscopic Sleeve Gastrectomy.

* p<0.05 12 months post-surgery compared to baseline.

** p<0.01 12 months post-surgery compared to baseline.

*** p<0.001 12 months post-surgery compared to baseline.

*is placed adjacent to the 12-month value to denote changes in RYGB or LSG respectively.

Data presented as mean (SD) or median (interquartile range). There were no statistically significant differences between cohorts (RYGB vs. LSG) pre-operatively. There were significant (p<0.05) reductions in the use of insulin and ACE-I/ARB, BMI, SBP, HbA1c, triglyceride and increase in HDL cholesterol (p<0.05) post-RYGB. There was a significant reduction in BMI and SBP whilst total cholesterol increased (p<0.05), most likely due to a 40% reduction in statin use post-LSG.

RYGB: Roux-en-Y Gastric Bypass; LSG: Laparoscopic Sleeve Gastrectomy; ACE-I: Angiotensin Converting Enzyme Inhibitors; ARB: Angiotensin II Receptor Blocker; BMI: Body Mass Index; BP: Blood Pressure; HbA1c: Glycated Haemoglobin; HDL-C: High Density Lipoprotein Cholesterol; LDL-C: Low Density Lipoprotein Cholesterol.
