# Supplementary material for: Improvements in Diabetic Neuropathy and Nephropathy After Bariatric Surgery: a Prospective Cohort Study
Source: Obes Surg. 2020 Oct 26;31(2):554–63. doi: 10.1007/s11695-020-05052-8 (PMC7847862; doi:10.1007/s11695-020-05052-8)
Supplement: Supplementary file 5 — (DOCX 17 kb) [file 11695_2020_5052_MOESM4_ESM.docx]

*Supplementary Table 3. Microvascular assessments pre and post-bariatric surgery.*

| Parameter | Baseline |  |  | 12 Months |  |  |
| --- | --- | --- | --- | --- | --- | --- |
|  | **RYGB** | **LSG** | **p** | **RYGB** | **LSG** | **p** |
| *Neuropathy Assessment* | | | | | | |
| NSP (x/38) | 3.7 (4.9) | 4.5 (2.1) | 0.477 | **0.53*** | **0.0*** | 0.323 |
| NDS (x/10) | 2 (2.5) | 1 (1.4) | 0.345 | 2 (3) | 1 (1) | 0.240 |
| VPT (volts) | 14 (7) | 15 (8) | 0.766 | 13 (7) | 18 (7) | 0.227 |
| CPT (°C) | 25.4 (20-28.2) | 27 (13.4-28.7) | 0.864 | 26.3 (22-28.3) | 24.5 (15.2-29.5) | 0.802 |
| WPT (°C) | 39.7 (4.3) | 41.4 (2.1) | 0.414 | 41.0 (5) | 42.9 (3.7) | 0.491 |
| DB-HRV (beats/min) | 17 (6) | 13 (2) | 0.295 | 16 (7) | 12 (2) | 0.257 |
| CNFD (no./mm^2^) | 26.0 (20-29) | 30.2 (21-32) | 0.379 | **28.9****  (25-32) | 34.4 (12-37) | 0.709 |
| CNBD (no./mm^2^) | 62.6 (38.1) | 66.3 (22.5) | 0.842 | **79**** (34.0) | 78.8 (45.2) | 0.735 |
| CNFL (mm/mm^2^) | 18.3 (16-23) | 22.2 (16-23) | 0.410 | **20.1**** (18-24) | 21.3 (17-26) | 0.767 |
| *Renal Assessment* | | | | | | |
| uACR (mg/mmol) | 1.06 (0.6-1.8) | 0.94 (0.5-11) | 1.00 | 0.50 (0.3-1) | 0.9 (0.3-6) | 0.483 |
| eGFR (ml/min) | 131 (28) | 122 (18) | 0.553 | **122**** (22) | 105 (26) | 0.191 |

Data are presented as mean (SD) or median (interquartile range).

There was a significant improvement in the NSP, CNFD, CNBD and CNFL (p<0.01). Other variables showed a non-significant trend towards improvement.

CNFD: Corneal Nerve Fibre Density; CNBD: Corneal Nerve Branch Density; CNFL: Corneal Nerve Fibre Length; NSP: Neuropathy Symptom Profile; NDS: Neuropathy Disability Score; VPT: Vibration Perception Threshold; CPT: Cold Perception Threshold; WPT: Warm Perception Threshold; DB-HRV: Deep Breathing Heart Rate Variability; ACR: albumin:creatinine ratio; sCreat: serum creatinine; sCysC: serum cystatin C; eGFR: estimated glomerular filtration rate.
